# Does coracoclavicular augmentation additional to hook plate fixation provide benefits in acute unstable acromioclavicular dislocation? A meta-analysis

**DOI:** 10.1186/s12891-022-05142-x

**Published:** 2022-03-04

**Authors:** Chih-Yao Lee, Po-Cheng Chen, Ying-Chun Liu, Yun-Che Tsai, Pei-Hsi Chou, Yin-Chih Fu, Wen-Chih Liu, Jesse Bernard Jupiter

**Affiliations:** 1 Department of Orthopedic Surgery, Kaohsiung Medical University Hospital, Kaohsiung Medical University, 807 Kaohsiung, Taiwan; 2 Department of Physical Medicine and Rehabilitation, Kaohsiung Chang Gung Memorial Hospital, College of Medicine, Chang Gung University, Kaohsiung, Taiwan; 3 Department of Public Health, College of Medicine, National Cheng Kung University, Tainan, Taiwan; 4 School of Post-Baccalaureate Medicine, College of Medicine, Kaohsiung Medical University, Kaohsiung, Taiwan; 5 Ph.D Program in Biomedical Engineering, College of Medicine, Kaohsiung Medical University, Kaohsiung, Taiwan; 6 Department of Orthopedic surgery, Kaohsiung Municipal Ta-Tung Hospital, Kaohsiug Medical University, Kaohsiung, Taiwan; 7 Department of Orthopedic Surgery, Kaohsiung Municipal Siaogang Hospital, Kaohsiung Medical University Hospital, Kaohsiung, Taiwan; 8 Hand and Arm center, Department of Orthopedic surgery, Massachusetts General Hospital, Boston, MA USA

**Keywords:** Acromioclavicular joint dislocation, Acromioclavicular joint separation, Hook plate, Coracoclavicular reconstruction, Coracoclavicular repair, Acromial osteolysis

## Abstract

**Background:**

Acromioclavicular joint (ACJ) dislocation is a common shoulder injury. In treating acute unstable ACJ dislocation, a hook plate (HP) is a straightforward and popular option for ensuring proper reduction and rigid fixation while promoting AC and coracoclavicular (CC) ligament healing. Surgeons typically remove the HP to prevent subacromial impingement and acromial osteolysis; however, concerns about redislocation after implant removal remain. Therefore, additional CC augmentation may be helpful in combination with HP fixation. The aim of this meta-analysis is to compare the outcomes and complications of HP fixation with or without additional CC augmentation for acute unstable ACJ dislocation.

**Methods:**

We searched the PubMed, EMBASE, and Web of Science databases for relevant case–control studies. The primary outcomes were patient-reported outcome measures; the secondary outcomes were pain measured using a visual analog scale (VAS), CC distance (CCD), and complications. Continuous data were assessed using weighted standardized mean differences (SMDs) with 95% confidence intervals (CIs), and dichotomous data were evaluated with Mantel–Haenszel odds ratio (ORs) with 95% CIs.

**Results:**

We analyzed one randomized control trial and four case–control studies comparing HP fixation with or without CC augmentation. A total of 474 patients with Rockwood type III or V ACJ dislocation were included. We found no differences in Constant–Murley score (SMD, − 0.58, 95% CI − 1.41 to 0.26; *P* = 0.18), American Shoulder and Elbow Surgeons score (SMD, 0.21, 95% CI − 0.10 to 0.52; *P* = 0.19), University of California at Los Angeles shoulder rating scale score (SMD, − 0.02, 95% CI − 1.27 to 1.23; *P* = 0.97), or VAS pain score (SMD, 0.36, 95% CI − 0.16 to 0.88; *P* = 0.17) between groups. The CC augmentation group had lower odds of osteolysis (OR, 0.27, 95% CI 0.10 to 0.74; *P* = 0.01) and a shorter CCD (SMD, − 0.29, 95% CI − 0.57 to − 0.01; *P* = 0.04).

**Conclusion:**

HP fixation with CC augmentation is preferable for acute unstable ACJ dislocations. Although CC augmentation did not provide additional benefits related to functional outcomes or pain, it resulted in greater reduction maintenance after implant removal and a 73% lower risk of acromial osteolysis.

**Trial registration:**

PROSPERO (CRD42021271118).

## Background

Acromioclavicular joint (ACJ) dislocation is a common injury that accounts for 50% of all sports-related shoulder injuries [1, 2]. The coracoclavicular (CC) and AC ligaments are the most important anatomical structures for maintaining the stability of the ACJ. Operative management is often indicated for Rockwood type III–VI ACJ dislocation [3], but operation for type III dislocation remains controversial [4–6].

Various treatment options for acute unstable ACJ dislocation, such as CC fixation (using Bosworth screws, suture anchors, hook plates [HPs], or TightRope) [3, 7–14], AC fixation (using tension band wires, Kirschner wires, or sutures) [8, 15], AC or CC reconstruction, and ligament transfer (Weaver–Dunn procedures) [16], have been investigated [17–22]. However, the optimal treatment for ACJ dislocation is still debated [3, 8, 23, 24]. The clavicle HP is used in one of the most common techniques to promote CC ligament scaring [25–27]. This device has many advantages, including facilitating a more straightforward surgical technique, rigid fixation, and early resumption of normal activities [25–27]. Despite these advantages, the HP should typically be removed after 3 to 6 months to prevent complications such as limited early shoulder motion, subacromial impingement, and acromial osteolysis [28–30]. Nevertheless, HP fixation is a safe and effective option for treating ACJ dislocation [31].

To lower the incidence of ACJ redislocation and the risk of acromial osteolysis, CC augmentation in conjunction with HP fixation is a reasonable option [27]. The possible benefits of combined HP fixation and CC augmentation include stronger initial fixation and support after removal of the HP. However, this combination’s efficacy remains unclear. The aim of this meta-analysis is to compare the outcomes and complications of HP fixation with and without CC augmentation in acute unstable ACJ dislocation. The primary outcomes were patient-reported outcome measures, and the secondary outcomes were a visual analog scale (VAS) score for pain, radiographically determined CC distance (CCD), the incidence of acromial osteolysis, and overall complications.

## Methods

### Search strategy and inclusion criteria

We conducted this study in accordance with the Preferred Reporting Items for Systematic Reviews and Meta-Analyses guidelines [32]. We searched three electronic databases, PubMed, Embase, and Web of Science up to July 27, 2021, using the following search string: (((acromioclavicular joint) OR (AC joint)) AND ((dislocation) OR (separation))) AND ((hook plate)). We reviewed the bibliographies of the resulting trials and related review articles manually for relevant references. Two independent reviewers (YCL and PCC) screened the titles and abstracts and examined the full texts of the eligible articles in detail. A third reviewer resolved any disagreements by making the final decision.

**Fig. 1:**
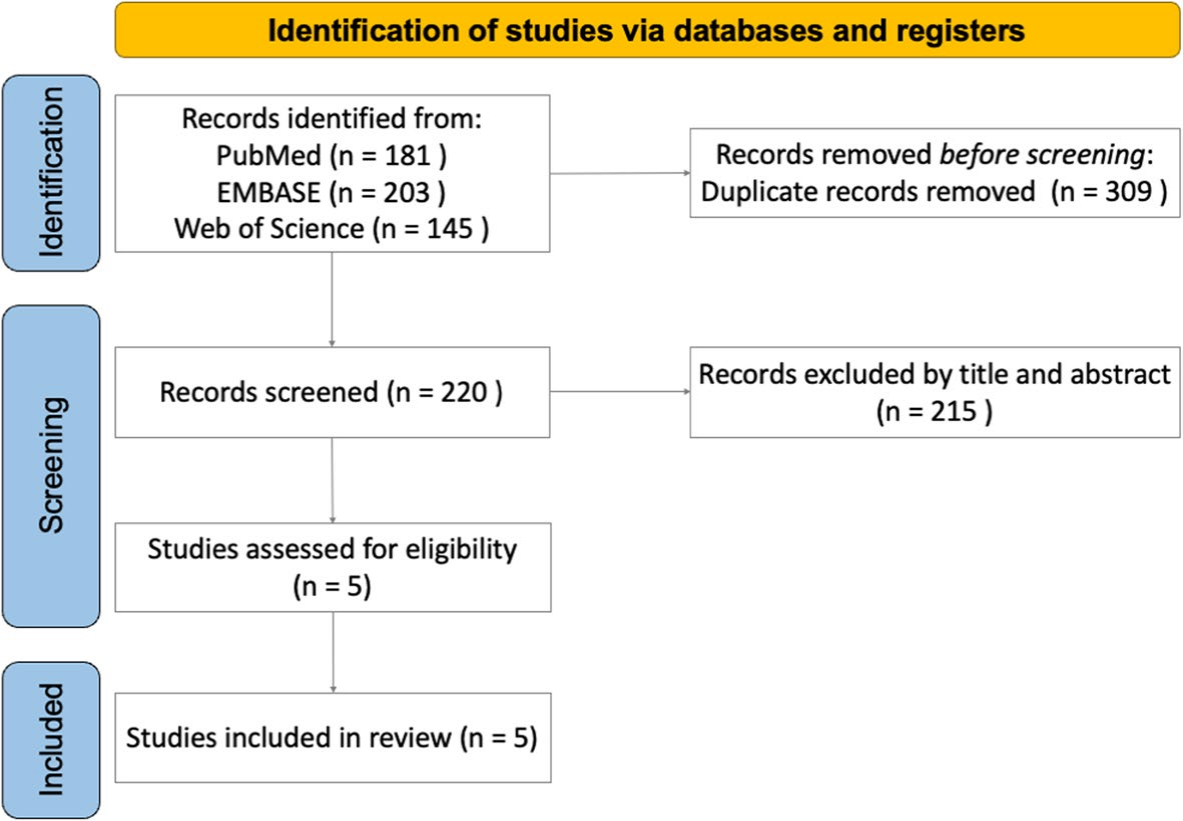
Preferred Reporting Items for Systematic Reviews and Meta-Analyses flow diagram of study search and screening for inclusion

We enrolled any prospective or retrospective case–control studies that met the following criteria: (1) a target population comprising patients with acute Rockwood type III, IV, or V ACJ displacement treated with HP fixation; (2) a comparative design with two treatment arms, one of HP fixation only and another of HP fixation with CC augmentation (CCHP); and (3) clinical outcomes measured at least 1 year after operation. We excluded (1) review articles and (2) duplicate publications. Fig. 1 visualizes the selection process.

### Methodological quality assessment

Two reviewers (YCL and YCT) assessed the quality of the studies independently, and the quality was confirmed by a third reviewer. We assessed the methodological quality of studies by using the Jadad scale for randomized controlled trials (RCTs) [33] and Newcastle–Ottawa scale (NOS) for prospective or retrospective comparative trials [34]. The Jadad scale is a 5-point scale that evaluates the methodologies of RCTs in three domains: randomization (2 points), blinding (2 points), and account of all patients (1 point) [33]. Higher scores indicate superior methodological quality. The NOS contains nine items measuring three domains: participant selection (four items, maximum of 4 points), comparability (one item, maximum of 2 points), and exposure (three items, maximum of 3 points). The maximal score is 9 points, with higher scores indicating superior methodological quality [34]. Cohen’s kappa coefficient was used to measure interrater reliability and to quantify the degree of agreement between the reviewers.

Discrepancies were resolved through discussion under the supervision of the corresponding author. In addition, we attempted to contact the primary authors of the included studies for any missing data.

### Data extraction

Two reviewers (CYL and PCC) independently extracted the following data related to the study characteristics and interventions and then verified the data together: study design, sample size, age distribution, sex distribution, follow-up period, types of HP, and suture used for CC fixation. The primary outcomes were the following patient-reported outcome measures: Constant–Murley score (CMS), the American Shoulder and Elbow Surgeons (ASES) shoulder score, and the University of California at Los Angeles (UCLA) shoulder scale score. The secondary outcomes were the VAS (0–10) score for pain, CCD, and complications (e.g., osteolysis, ACJ osteoarthritis, peri-implant fracture, and infection).

### Meta-analysis methodology

The senior author (WCL) used Comprehensive Meta-Analysis version 3 (Biostat, Englewood, NJ, USA) to pool the effects of interest from our included studies.

For continuous data (primary outcomes, CCD, and VAS score), we estimated the summary of standardized mean differences (SMDs) and 95% confidence intervals (CIs). For dichotomous data (complications), we estimated the odds ratio (OR) and 95% CI. We used the Mantel–Haenszel (MH) method to estimate the effect size for binary outcomes and the inverse variance method to pool the effect size for continuous results. A negative SMD indicated that CCHP was the more favorable treatment option. ORs of < 1 and > 1 respectively indicated CCHP and HP fixation alone to be the preferable treatment option. We used a Cochran Q test to evaluate heterogeneity. A two-tailed *P* value < 0.01 was considered statistically significant. We approximated the percentage of variability in the heterogeneity estimation by using *I*^2^ [35]. We employed subgroup analyses to determine the sources of heterogeneity. We employed random-effects models to calculate all the relationships. We employed Egger test [36] and funnel plots [37] to assess publication bias.

## Results

### Literature search and study characteristics

We reviewed the titles and abstracts of 220 unique articles; five articles met the inclusion criteria and were assessed further for eligibility. No studies were excluded on the basis of the eligibility assessment (Fig. 1). Ultimately, we obtained one RCT [38] and four retrospective case–control studies [39–42] for the eventual meta-analysis.

A total of 474 patients were included, and we broke down the numbers of patients, mean ages, and sex ratios of the comparator groups. All studies reported patient sex and age data. Four studies enrolled patients with Rockwood type III or V ACJ dislocation; one study enrolled type V dislocation only [41]. Two studies enrolled patients who had experienced traumatic injury less than 2 weeks prior to study commencement [39, 41, 42], one enrolled patients who had experienced traumatic injury less than 4 weeks prior to study commencement [40], and one enrolled patients who had experienced traumatic injury less than 6 weeks prior to study commencement [38]. One RCT [38] received Jadad score of 3. Four retrospective case–control studies were evaluated using the NOS; one received a score of 8 [40], and three received scores of 7 [39, 41, 42]. Table 1 presents the details of each study. The degree of agreement between reviewers on the NOS and Jadad scores is reported in Table 2.

**Table 1:** Details of studies on hook plate fixation with or without coracoclavicular augmentation for acromioclavicular dislocation

Study	Study design	Treatment	Sex (M/F)	Rockwood type (III/ V)	Injury to Surgery	Age (y)^a^	Time for implant removal^a^	Follow-up (m)^a^	Outcome measures	Quality assessment
**Yin 2018** **[**38**]**	RCT	HP only	18/7	16/10	< 6 weeks	44.5 ± 12.3	12.9 ± 1.8 mo	21.3 ± 2.0	VAS/ASES/CMS/Karlsson score/ patient satisfaction	3^b^
		CCHP	20/6	14/11		46.3 ± 11.9	4.9 ± 1.2 mo	19.9 ± 2.9		
**Chang 2019** **[**39**]**	RCS	HP only	16/10	12/14	< 2 weeks	50 (24–69)	6 (3–12) mo	11 (9–27.1)	VAS/UCLA/ASES/Return to work	7^c^
		CCHP	15/6	7/14		44 (21–81)	6 (5–10) mo	9.5 (9–11.4)		
**Liu 2020** **[**41**]**	RCS	HP only	–	0/112	< 2 weeks	46 (19–75)	102.8 (89–116) d	6 (after implant removal)	VAS/CMS	7^c^
		CCHP	–	0/105		50 (18–62)	101.3 (86–118) d			
**Seo 2020** **[**42**]**	RCS	HP only	38/9	26/21	< 2 weeks	44.6 ± 15.4	4.2 ± 0.7 mo	18.7 ± 6.2	ASES/KSS/CMS	7^c^
		CCHP	65/8	34/39		46.9 ± 12.6	4.0 ± 1.0 mo	18.1 ± 4.8		
**Chen 2021** **[**40**]**	RCS	HP only	15/4	9/10	< 4 weeks	44.5 ± 15.4	5.3 ± 1.5 mo	38.5 ± 24.9	VAS/CMS/UCLA/Taft score	8^c^
		CCHP	13/6	10/9		46.4 ± 16.4	5.7 ± 1.6 mo	2.6 ± 21.7		

*RCS* retrospective case–control study, *RCT* randomized controlled trial, *CCHP* coracoclavicular augmentation with hook plate fixation, *HP* hook plate, *mo* month, *d* day, *VAS* visual analog scale score for pain, *UCLA* University of California at Los Angeles shoulder rating scale score, *ASES* American Shoulder and Elbow Surgeons score, *KSS* Korean shoulder score, *CMS* Constant–Murley score

^a^Mean ± standard deviation and Mean (range) are given for these variables

^b^Assessed using the Jadad scale

^c^Assessed using the Newcastle–Ottawa scale

**Table 2:** Interrater reliability of Newcastle–Ottawa and Jadad scales between two reviewers

	Inter-rater reliability
	Kappa coefficient (95% CI)
**Newcastle–Ottawa scale (4 studies)**	
**Selection**	
Is the case definition adequate	1
Representativeness of the cases	1
Selection of Controls	1
Definition of Controls	1
**Comparability**	
Study control	0.20 (−0.27 to 0.67)
Any additional factor	1
**Outcome**	
Assessment of outcome	1
Was follow-up long enough for outcomes to occur	1
Adequacy of follow up of cohorts	0.50 (−0.24 to 1.00)
**Jadad scale (1 study)**	
**Randomization**	1
**Blinding**	1
**Withdrawal and dropouts**	1

### Summary of surgical procedures and postoperative management

In the HP fixation groups of all included studies, all ACJs underwent debridement before reduction. In two studies, the AC ligament was repaired before placement of the HP—one with an absorbable suture [38] and the other with tape fixation [39]. The other studies did not mention any repair of the AC ligament [40–42]. After the dislocated ACJ was reduced, the HP was inserted posterior to the ACJ and fixed the clavicle with screws. In the CCHP group, the ACJs were prepared and reduced as in the HP treatments. The CC augmentation consisted of either repair or reconstruction of the CC ligament before HP fixation. In one study, the CC ligament was repaired with an absorbable Vicryl No. 1 suture (Ethicon, Cincinnati, OH, USA) [42]. The materials used for CC augmentation differed. In one study, 6-mm nylon was used to reconstruct the CC ligament through bone tunnel on clavicle [41]; the CC ligament was reconstructed with a sterile polyester surgical suture (Mersilene Polyester Fiber Suture, Ethicon, Cincinnati, OH, USA) in two studies [39, 40]; one through two tunnels on clavicle [40] and the other make multiple knots at the anterior to the clavicle [39]. One study, double-tunnel CC ligament reconstruction was performed with an autograft harvested from the lateral half of the short head of the biceps tendon [38]. All HPs were removed after 3 to 6 months of fixation except for those in the HP only group in one study, which were removed after 12.95 months [38].

### Patient-reported outcome measures

**Fig. 2:**
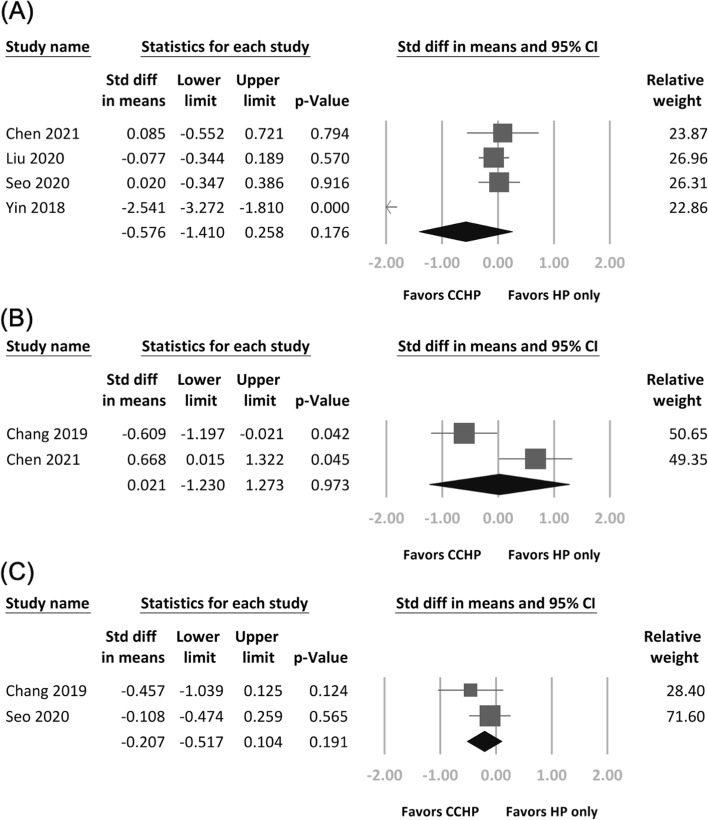
Forest plots of the weighted standardized mean differences in (**A**) Constant–Murley score, (**B**) University of California at Los Angeles shoulder scale score, and (**C**) American Shoulder and Elbow Surgeons score between treatment arms

No significant differences were identified in CMS (*N* = 427; SMD, − 0.58, 95% CI − 1.41 to 0.26; *P* = 0.18; Fig. 2a), UCLA score (*N* = 85; SMD, 0.02, 95% CI − 1.23 to 1.27; *P* = 0.97; Fig. 2b), or ASES score (*N* = 167; SMD, − 0.21, 95% CI − 0.52 to 0.10; *P* = 0.19; Fig. 2c). Heterogeneity was present in the CMS (*I*^2^ = 93%) and UCLA score (*I*^2^ = 88%) results but not in the ASES score results (*I*^2^ = 0%).

### Pain VAS scores

**Fig. 3:**
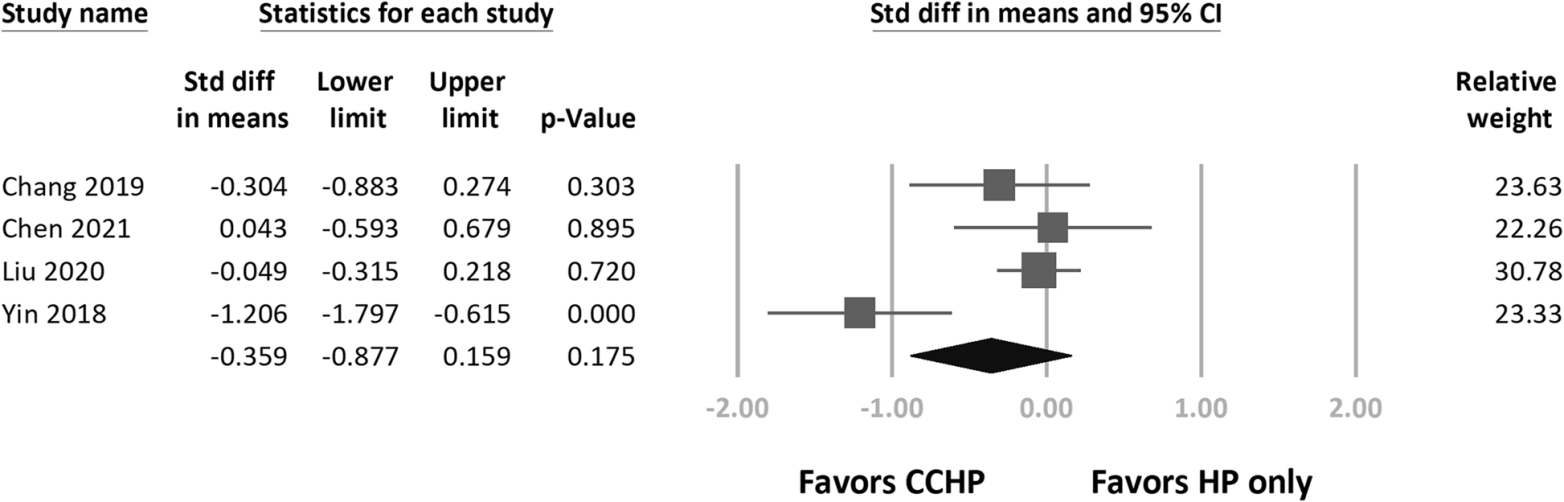
Forest plots of weighted standardized mean differences in visual analog scale (VAS) score for pain between treatment arms

VAS pain scores did not differ significantly (*N* = 354; SMD, 0.36, 95% CI − 0.16 to 0.88; *P* = 0.17; Fig. 3). Heterogeneity was present in the VAS results (*I*^2^ = 77%).

### CCD

**Fig. 4:**
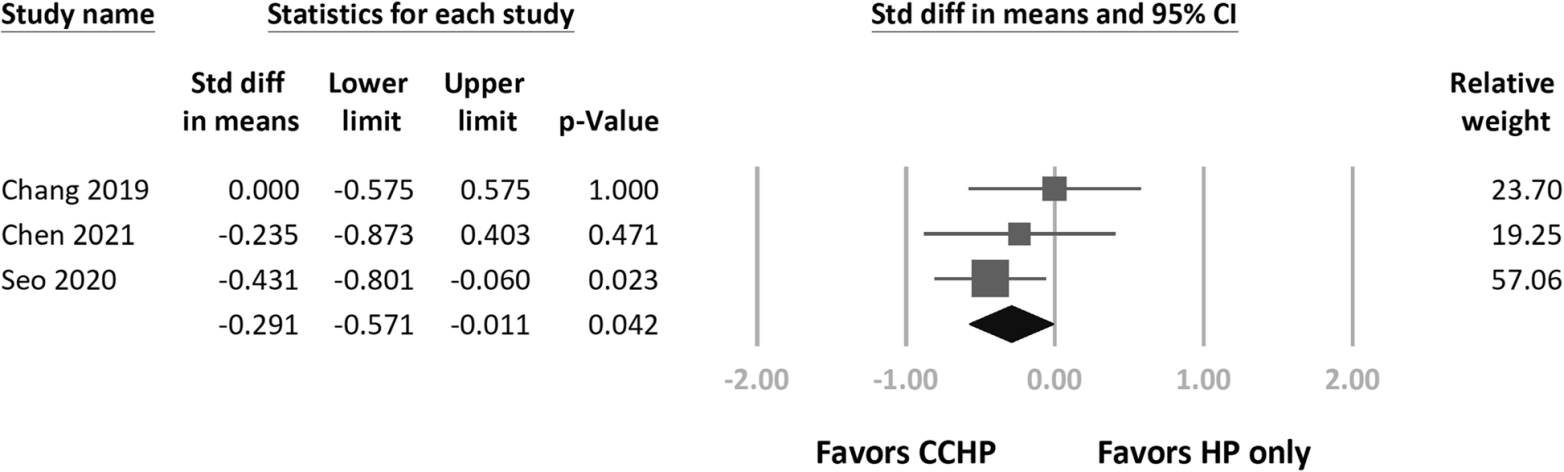
Forest plots of weighted standardized mean differences in coracoclavicular distance between treatment arms

CCD significantly differed between groups (*N* = 205; SMD, − 0.29, 95% CI − 0.57 to − 0.01; *P* = 0.04; Fig. 4), suggesting superior CCD maintenance in the CCHF group. No heterogeneity was present in the CCD results (*I*^2^ = 0%).

### Complications such as acromial Osteolysis

**Fig. 5:**
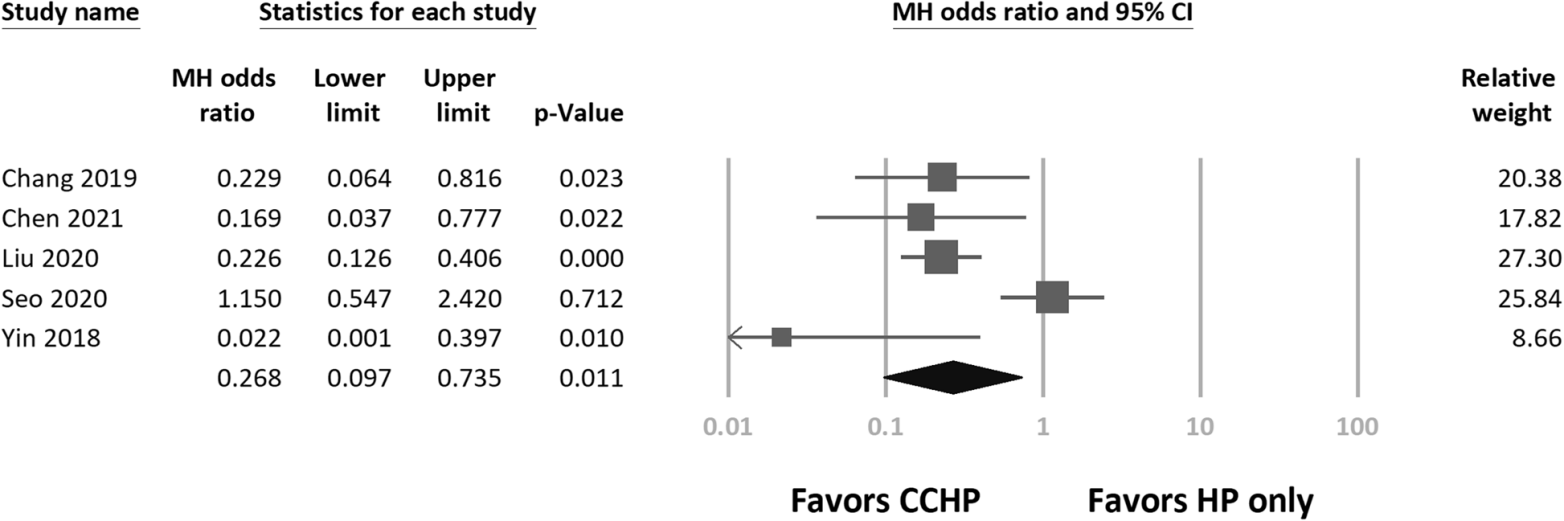
Forest plots of the Mantel–Haenszel odds ratios for acromial osteolysis between treatment arms

All complications in each included study are listed in Table 3. Among all complications, we found significantly greater odds of acromial osteolysis (*N* = 474; odds ratio (OR), 0.27, 95% CI 0.10 to 0.74; *P* = 0.01; Fig. 5) for the HP only group. Heterogeneity was present in the acromial osteolysis results (*I*^2^ = 76%). Comparing only the studies with early implant removal (< 6 months) in both the HP only and CCHP groups [39–42], we still found more acromial osteolysis in the HP only group (*N* = 420; MH OR, 0.34, 95% CI 0.13 to 0.92; *P* = 0.03).

**Table 3:** Details of complications in each study

Study	Complications	
	CCHP	HP only
**Yin 2018**	Acromial osteolysis (0/26)	Acromial osteolysis (12/25)
**Chang 2019**	Acromial osteolysis (5/21) ACJ arthrosis (3/21)	Acromial osteolysis (15/26) ACJ arthrosis (8/26) Superficial wound infection (1/26)
**Liu 2020**	Acromial osteolysis (25/105) Superficial wound infection (2/105) Peri-implant fracture (1/105)	Acromial osteolysis (65/112) Peri-implant fracture (8/112)
**Seo 2020**	Acromial osteolysis (32/73) ACJ arthrosis (16/73) Stiffness before implants removal (25/73)	Acromial osteolysis (19/47) ACJ arthrosis (12/47) Stiffness before implants removal (16/47)
**Chen 2021**	Acromial osteolysis (10/19) Distal clavicle osteolysis (2/19)	Acromial osteolysis (3/19) Distal clavicle osteolysis (1/19)

*CCHP* coracoclavicular augmentation and hook plate fixation, *HP* hook plate, *ACJ* acromioclavicular joint

### Subgroup analysis

**Fig. 6:**
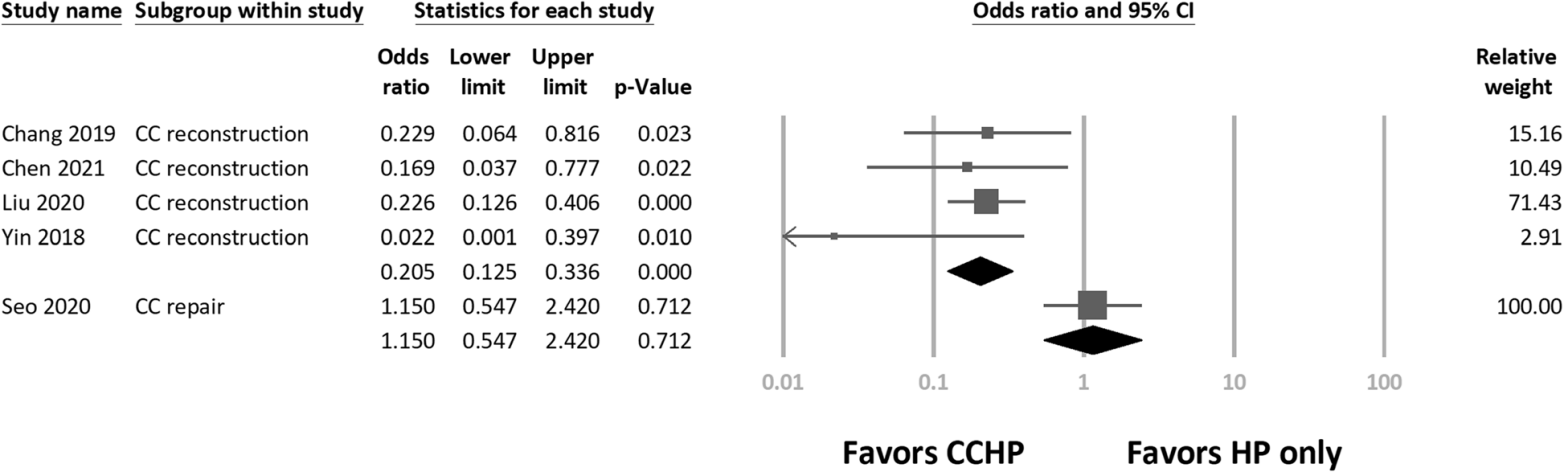
Forest plots of the Mantel–Haenszel odds ratios for acromial osteolysis between coracoclavicular (CC) reconstruction and CC repair

We subdivided CC augmentation into CC ligament reconstruction [38–41] and CC ligament repair [42] groups. CCD did not differ between groups (Q = 1.27, *df* = 1, *P* = 0.259). Although the incidence of acromial osteolysis differed significantly from that of HP fixation alone (Q = 14.3, *df* = 1, *P* < 0.01), no difference was found between the CC reconstruction and repair subgroups (Q = 2.49, *df* = 3, *P* = 0.48; Fig. 6).

### Publication Bias

**Fig. 7:**
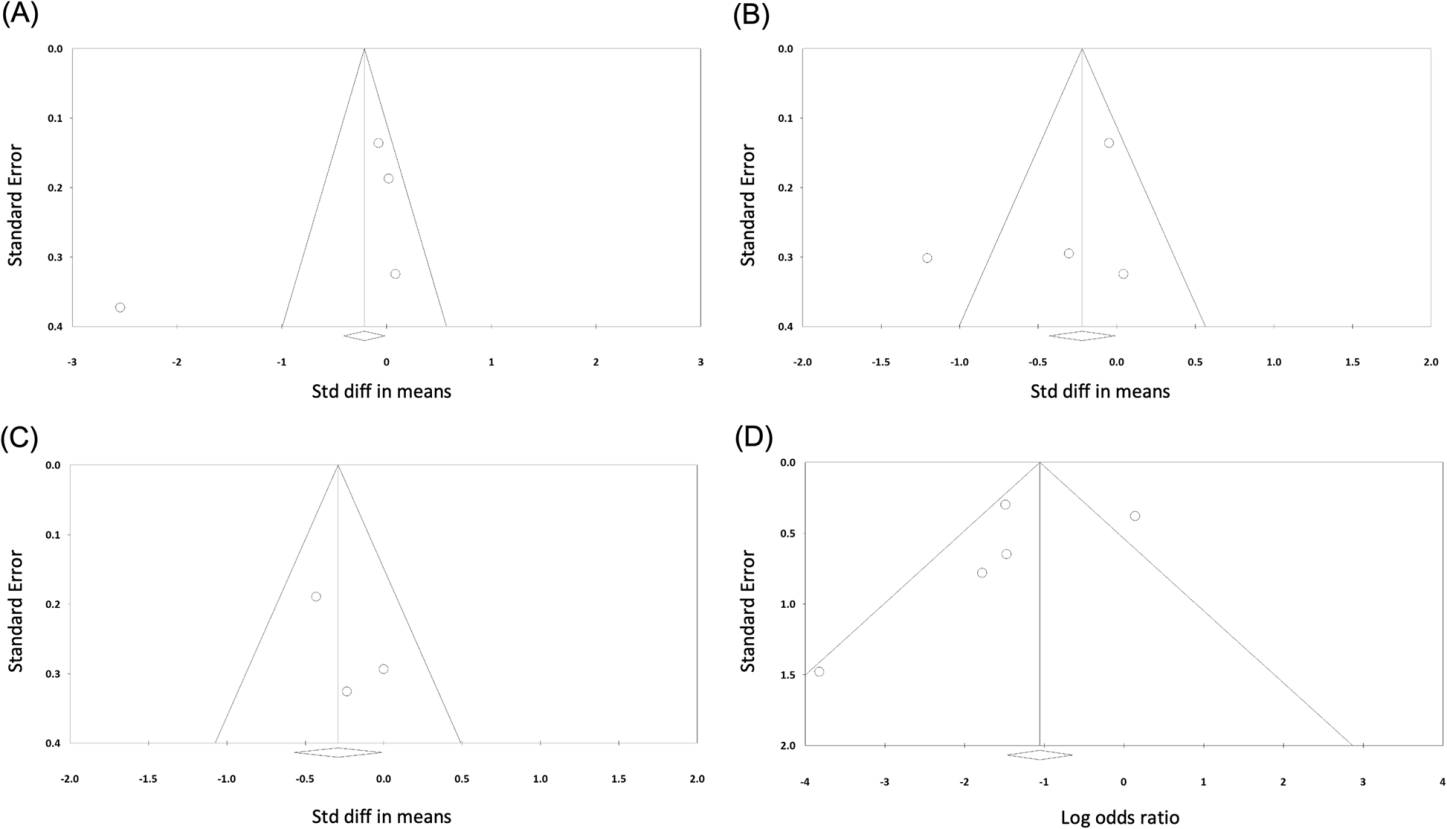
Funnel plots for (**A**) Constant–Murley score, (**B**) visual analog scale score, (**C**) coracoclavicular distance, and (**D**) incidence of acromial osteolysis measures

Because at least three of the studies could run publication bias procedures for an outcome, we could not evaluate publication bias for the UCLA or ASES score The Egger test revealed no significant publication bias for the CMS, VAS, CCD, or acromial osteolysis measures (Table 4); the funnel plots for the CMS, VAS, CCD, and acromial osteolysis measures are symmetric (Fig. 7).

**Table 4:** Egger test results for each outcome

	***t***-value	***df***	***P*** value
CMS	1.15	2	0.37
VAS	0.89	2	0.47
CCD	1.40	1	0.39
Acromial osteolysis	0.80	3	0.48

*CMS* Constant–Murley score, *VAS* visual analog scale score for pain, *CCD*

## Discussion

This meta-analysis included five case–control studies enrolling patients with acute Rockwood type III or V ACJ dislocation and comparing HP fixation with and without CC augmentation. The CC augmentation group consisted of patients who had undergone CC ligament repair or CC reconstruction with artificial surgical tape or autograft. We found that CCHP showed no advantage over HP alone in functional outcomes or pain; however, CCHP resulted in superior CCD maintenance and a lower incidence of acromial osteolysis.

Acromial osteolysis is one of the most common complications of HP fixation for ACJ dislocation treatment and is caused by the high pressure between the acromion and hook [43]. Acromial osteolysis may further result in chronic shoulder pain and impaired functional outcomes [18, 30]. In the HP only group, the stability of the ACJ relied mainly on the scar tissue at the base of the CC ligament. However, such scar tissue can rupture easily, resulting in redislocation under an uneven stress distribution. Therefore, concomitant CC augmentation to share the load from the acromion to the coracoid and clavicle is a viable option. A shorter CCD implies superior maintenance of AC reduction after implant removal in CCHP group. However, the CCHP group did not achieve more favorable functional outcomes and lower pain scores. The patient-reported outcome measures were physical function, psychosocial issues, and quality of life [44]. Therefore, superior CCD maintenance does not necessarily lead to superior patient-reported outcomes; this result is consistent with that of a previous study [45].

Heterogeneity was identified in CMSs (*I*^2^ = 93%), UCLA scores (*I*^2^ = 88%), VAS scores (*I*^2^ = 77%), and osteolysis incidence rates (*I*^2^ = 76%). This heterogeneity might be attributable to several factors, such as the intervals between injury and surgery, CC augmentation methods, and HP removal time. All the studies included patients with acute unstable ACJ dislocation; however, the rates of injury after trauma varied. The AC and CC ligaments are considered to lose their potential to heal by 3 weeks after trauma [46, 47], and the definition of chronic ACJ dislocation is injury persisting for more than 6 weeks after trauma [48]. The definition of such injuries between 3 to 6 weeks after trauma is uncertain. One of the included studies enrolled patients who had experienced traumatic injury less than 4 weeks prior to study commencement [40], and one enrolled patients who had experienced traumatic injury less than 6 weeks prior to study commencement [38]. The other studies enrolled patients who had experienced traumatic injury less than 2 weeks prior to study commencement [39, 41, 42]. Due to the limited number of included studies, we could not perform subgroup analysis to eliminate the heterogeneity in the interval between injury and surgery.

CC augmentation can be performed through CC ligament repair or reconstruction. Four studies [38–41] reported on CC ligament reconstruction; the other reported on CC ligament repair [42]. In subgroup analysis, CC ligament reconstruction was associated with a lower incidence of osteolysis than HP alone, but CC ligament repair was not. We found a significant difference between the subgroup of CC ligament reconstruction and CC repair (Q = 14.3, *P* < 0.01). In addition, we found no differences within the CC reconstruction group, which implied that suture tape and autologous tendon result in a similar acromion load-sharing effect and reduce the odds of osteolysis. However, we found no difference between groups in CCD (Q = 1.27, *P* = 0.259). Thus, both CC ligament repair and reconstruction for CCHP can result in superior CCD maintenance than HP alone can.

The CCHP group had 73% less acromial osteolysis than the HP only group. In one study [38], the implants in the HP only group were removed after 12.9 months, which is over 6 months longer than the period used by other the HP only groups. The different duration of HP fixation might have confounded the results regarding acromial osteolysis. Therefore, we compared only the studies with similar implant removal times between the two groups [39–42]. We found that the CCHP group was still 66% less likely to develop acromial osteolysis.

Some limitations in this study were as follows. This meta-analysis comprised only one RCT and four retrospective, nonrandomized case–control studies. We should consider potential selection bias in retrospective studies; however, the characteristics of the included patients were similar between the two groups. In addition, measurement bias existed, especially in those studies in which CC reconstruction was performed with a transosseous tunnel in the clavicle. Due to few included studies and short-term follow-ups, we could not perform subgroup analysis to determine the heterogeneity of each outcome.

## Conclusion

On the basis of our analytical results, we favor additional CC augmentation in combination with HP fixation for treating acute and unstable ACJ dislocations. Although no superiority was demonstrated in functional outcomes or pain, additional CC augmentation resulted in superior reduction maintenance after implant removal and a 73% lower risk of acromial osteolysis. We recommend further research target patients prone to acromial osteolysis and monitor their long-term functional and clinical outcomes to validate our findings.

## Data Availability

The datasets used/or analyzed during the current study are available from the corresponding author on reasonable request.

## Abbreviations

ASES: American Shoulder and Elbow Surgeons score
CC: Coracoclavicular
CCD: Coracoclavicular distance
CCHP: Coracoclavicular augmentation with hook plate fixation
CI: Confidence interval
CMS: Constant–Murley score
HP: Hook plate
MH: Mantel-Haenszel
OR: Odds ratio
SMD: Standardized mean difference
UCLA: University of California at Los Angeles shoulder rating scale
